# Intelligence

**Published:** 1831-10

**Authors:** 


### INTELLIGENCE.

### KING'S COLLEGE, LONDON.

The ceremony of opening King's College will take place on Saturday, the 8th of October,* when the Bishop of London will deliver an inaugural discourse. The Medical School will open on the following Monday, and the senior and junior departments on Monday, the 17th October.

---

### MIDDLESEX HOSPITAL.

Mr. Arnott has been elected Assistant Surgeon to this institution.

---

### ROYAL COLLEGE OF SURGEONS.

Mr. Mayo has resigned the junior professorship of Anatomy and Surgery at the Royal College of Surgeons, which he has held during the last three years. Mr. Brodie held this situation for the same period; Mr. Lawrence for four years; Mr. Green, we believe, for five. Mr. Mayo is succeeded by Mr. Charles Bell.

---

Dr. Charles Mansfield Clarke, Physician to her Majesty, has recently been created a baronet. We are sure that the profession in general will be gratified that such a mark of distinction has been conferred upon Dr. Clarke: his professional ability, and his invariable liberality and kindness to all classes of his brethren, have obtained for him their universal esteem. We are not fond of indulging in eulogies upon the living; "they smack of flattery;" but in this instance we are safe from any such imputation, for we only echo the general voice.

---

### BOARD OF HEALTH.

*Preliminary Steps advised to be taken on the first Appearance of Cholera.*

It is of great importance that each town or village, particularly those on the coast, should be prepared with the best arranged means to meet such a calamity as the breaking out of the disease now raging in the North of Europe, so as to prevent confusion upon the emergency of the moment, and be ready to act upon a well-considered system for preventing the spreading of infection.

With this view the Board recommends the formation of a local board of health at each place, to consist of the chief magistrate, the clergyman, one or more medical gentlemen, and two or three of the principal inhabitants, who may immediately, and as occasion requires, correspond with the Board of Health in London, the medical members of the local Board being deputed to write upon all subjects relating to any symptoms of the disease.

The best means of preventing the spreading of infection are, the immediate separation of the uninfected from the sick, by their prompt removal from the house of any infected person, or by the removal of any individual affected with the disease, if possible, to some house in a dry and airy situation appropriated to the purpose; but in the event of such removal not being practicable, on account of extreme illness or otherwise, the prevention of all intercourse with the sick, even of the family of the person attacked, must be rigidly observed, unless the individuals who desire to stay shall submit to such strict rules of quarantine as the public safety may demand, and the local Board of Health, advising with the Board of Health in London, may consider expedient.

As success in the treatment of this disease, and preventing it spreading, have

* It was stated, by mistake, in our last number, that King's College would open on the 8th November.—Ed.

been found greatly to depend upon early medical assistance, it is of great importance that the heads of families and others should be vigilant in guarding against concealment or delay in making known every case which may occur.

On the removal of diseased persons, the rooms they may have inhabited, and the house generally, should be thoroughly exposed to a constant current of air, and recourse had to all the well-known means of purifying houses, particularly the use of chloride of lime; and the bedding and clothing of the sick person, after removal, should be soaked in a slight solution of the chloride in water, and well washed. It is impossible that ventilation and cleanliness can be carried too far in the houses of the sick after removal; whitewashing, and a variety of means of effecting so important an object, will no doubt occur to the local Boards of Health, and a continuance of ventilation for some days, as the best means of preventing contagion.

In large towns, the local Board of Health should be composed of sufficient numbers to admit of subdivision into district committees, always attaching to each committee at least one medical gentleman.

For the information of the public, and to secure a ready and instant reference to authorized persons, the names and residence of the persons composing the local Boards of Health should be placed on the church door.

In the event of so great a calamity falling upon this country as the introduction of this disease, rules and regulations upon an extensive scale, suited to the rigid system of quarantine which such an event would demand, will be immediately circulated by the Lords of his Majesty's Most Honourable Privy Council, who will, upon the earliest intimation of the existence of the disease, send down a medical practitioner, who has been acquainted with the disease as it occurred in India.

In the name of the Board,

HENRY HALFORD, President.

---

*Description of the Cholera Morbus, by* Drs. Barry *and* Russell.

We take the following account of the cholera morbus from a pamphlet recently published by the Board of Health.

*Extract from the joint Report of Drs. Russell and Barry.*

*St. Petersburgh;* 27 (15) *July,* 1831.

Sir: Although there can be no doubt that the disease now prevailing here is strictly identical, in all essential points, with the epidemic cholera of India; and although there are many descriptions extant of that malady, much more ably and accurately drawn up than any which we can pretend to give; yet we are induced to believe that a short account of the symptoms which we ourselves have actually witnessed and noted at the bedside in some hundreds of cases, since our arrival here, may be useful; first, because we are not aware that any description by an eyewitness of European cholera has yet been addressed to the British government; secondly, because the disease, as it has shewn itself in this capital, when closely compared with the Indian cholera, appears to have undergone some modifications; thirdly, because, having now studied the disease in all its stages, our description, however imperfect, will at least assist towards establishing a standard of comparison with other local epidemics of cholera in Europe, and may, perhaps, enable those who have not seen this disease, to recognise it with more certainty than they would otherwise be able to do.

The cholera morbus of the north of Europe, to which the Russian peasants have given the name of "chornaia colezn," or black illness, like most other diseases, is accompanied by a set of symptoms which may be termed preliminary; by another set which strongly mark the disease, in its first, cold, or collapse stage; and by a third set, which characterise the second stage, that of reaction, heat, and fever.

*Preliminary Symptoms.* We have but few opportunities of witnessing the presence of all these symptoms, some of which precede the complete seizure by so short an interval, that the utmost diligence is scarcely sufficient to bring the patient and the physician together, after their occurrence, before the disease is fully formed. Diarrhœa, at first feculent, with slight cramps in the legs, nausea, pain, or heat about the pit of the stomach, malaise, give the longest warning. Indeed, purging, or ordinary diarrhœa, has been frequently known to continue for one, two, or more days, unaccompanied by any other remarkable symptom, until the patient is suddenly struck blue, and nearly lifeless. Often the symptoms just mentioned are arrested by timely judicious treatment, and the disease completely averted. When violent vertigo, sick stomach, nervous agitation, intermittent, slow, or small pulse, cramps, beginning at the tip of the fingers and toes, and rapidly approaching the trunk, give the first warning, then there is scarcely an interval. Vomiting or purging, or both these evacuations, of a liquid like ricewater or whey, or barley water, come on; the features become sharp and contracted, the eye sinks, the look is expressive of terror, wildness, and, as it were, a consciousness on the part of the sufferer that the hand of death is upon him. The lips, the face, the neck, the hands, the feet, and soon the thighs, arms, and whole surface, assume a leaden, blue, purple, black, or deep brown tint, according to the complexion of the individual, varying in shade with the intensity of the attack. The fingers and toes are reduced at least a third in thickness; the skin and soft parts covering them are wrinkled, shrivelled, and folded; the nails put on a bluish pearl white; the larger superficial veins are marked by flat lines of a deeper black; the pulse is either small as a thread, and scarcely vibrating, or else totally extinct. The skin is deadly cold, and often damp; the tongue always moist, often white and loaded, but flabby and chilled, like a bit of dead flesh. The voice is nearly gone; the respiration quick, irregular, and imperfectly performed. Inspiration appears to be effected by an immense effort of the chest, whilst the alæ nasi (in the most hopeless cases, and towards their close), instead of expanding, collapse, and stop the ingress of air. Expiration is quick and convulsive. The patient asks only for water, speaks in a plaintive whisper (the 'vox cholerica,') and only by a word at a time, from not being able to retain air enough in his lungs for a sentence. He tosses incessantly from side to side, and complains of intolerable weight and anguish around his heart. He struggles for breath, and often lays his hand on his stomach and chest to point out the seat of his agony. The integuments of the belly are sometimes raised into high irregular folds, whilst the belly itself is violently drawn in, the diaphragm upwards and inwards, towards the chest; sometimes there are tetanic spasms of the legs, thighs, and loins; but we have not seen general tetanus, nor even trismus. There is occasionally a low suffering whine. The secretion of urine is always totally suspended, nor have we observed tears shed under these circumstances; vomiting and purging, which are far from being the most important or dangerous symptoms, and which, in a very great number of cases of the present epidemic, have not been profuse, generally cease, or are arrested by medicine easily in the attack. Frictions remove the blue colour for a time from the part rubbed; but in other parts, particularly the face, the livor becomes every moment more intense and more general. The lips and cheeks sometimes puff out and flap, in expiration, with a white froth between them, as in apoplexy. If blood be obtained in this state, it is black, flows by drops, is thick, and feels to the finger colder than natural. Towards the close of this scene, the respiration becoms very slow, there is a quivering among the tendons of the wrist; the mind remains entire. The patient is first unable to swallow, then becomes insensible; there never is, however, any rattle in the throat, and he dies quietly, after a long convulsive sob or two.

The above is a faint description of the very worst kind of case, dying, in the cold stage, in from six to twenty-four hours after the setting in of the bad symptoms. We have seen many such cases just carried to the hospital from their homes or their barracks. In by far the greater number, vomiting had ceased: in some, however, it was still going on, and invariably of the true serous kind. Many confessed that they had concealed a diarrhœa for a day or two; others had been suddenly seized, generally very early in the morning.

From the aggravated state which we have just described, but very few indeed recover, particularly if that state has been present even for four hours before treatment has commenced. A thread of pulse, however small, is almost always felt at the wrist, where recovery from the blue or cold stage is to be expected. Singular enough to say, hiccough coming on in the intermediate moments, between the threatening of death and the beginning of reaction, is a favorable sign, and generally announces the return of circulation.

In less severe cases, the pulse is not wholly extinguished, though much reduced in volume; the respiration is less embarrassed; the oppression and anguish at the chest are not so overwhelming, although vomiting and purging and the cramps may have been more intense. The coldness and change of colour of the surface, the peculiar alteration of the voice, a greater or less degree of coldness of the tongue, the character of the liquids evacuated, have been invariably well marked in all the degrees of violence of attack which we have hitherto witnessed in this epidemic. In no case or stage of this disease have we observed shivering; nor have we heard, after inquiry, of more than one case, in which this febrile symptom took place.

*Fever, or Hot Stage.* After the blue cold period has lasted from twelve to twenty-four, seldom to forty-eight hours or upwards, the pulse and external heat begin gradually to return; headach is complained of, with noise in the ears; the tongue becomes more loaded, redder at the tip and edges, and also drier. High-coloured urine is passed with pain and in small quantities, the pupil is often dilated, soreness is felt on pressure over the liver, stomach, and belly; bleeding by the lancet or leeches is required; ice to the head gives great relief; in short, the patient is now labouring under a continued fever, not to be distinguished from ordinary fever. A profuse critical perspiration may come on from the second or third day, and leave the sufferer convalescent; but, much more frequently, the quickness of pulse and heat of skin continue; the tongue becomes brown and parched, the eyes are suffused and drowsy, there is a dull flush, with stupor and heaviness about the countenance, much resembling typhus; dark sordes collect about the lips and teeth; sometimes the patient is pale, squalid, and low, with the pulse and heat below natural, but with the typhus stupor; delirium supervenes, and death takes place from the fourth to the eighth day, or even later, in the very individual, too, whom the most assiduous attention had barely saved in the first or cold stage. To give a notion of the importance and danger of cholera fever, a most intelligent physician, Dr. Reimer, of the merchant hospital, informs us, that of the twenty cases treated under his own eye, who fell victims to the disease, seven died in the cold stage, and thirteen in the consecutive fever.

The singular malady is only cognizable with certainty during its blue or cold period. After reaction has been established, it cannot be distinguished from an ordinary continued fever, except by the shortness and fatality of its course. The greenish or dark, and highly-bilious, discharges produced in the hot stage, by calomel, are not sufficiently diagnostic; and it is curious that the persons employed about these typhoid cases, when they are attacked, are never seized with ordinary fever, but with genuine cold blue cholera. Nothing, therefore, is more certain, than that persons may come to the coast of England, apparently labouring under common feverish indisposition, who really and truly are suffering under cholera in the second stage.

The points of difference between the present epidemic and the cholera of

India, when the two diseases are closely compared, appear to us to be the following:

First. The evacuations, both upwards and downwards, seem to have been much more profuse and ungovernable in the Indian than in the present cholera, though the characters of the evacuations are precisely the same.

Secondly. Restoration to health from the cold stage, without passing through consecutive fever of any kind, was by far more frequent in India than here: nor did the consecutive fever there assume a typhoid type.

Thirdly. The proportion of deaths in the cold stage, compared with those in the hot, was far greater in India according to Dr. Russell's experience, than here.

Fourthly. The number of medical men and hospital attendants attacked with cholera during the present epidemic, in proportion to the whole employed and to the other classes of society, has been, beyond all comparison, greater here than in India under similar circumstances: twenty-five medical men have been already seized, and nine have died out of two hundred and sixty-four. Four others have died at Cronstadt, out of a very small number residing in that fortress at the time the disease broke out there. Six attendants have been taken ill at a small temporary hospital behind the Aboucoff since we wrote last. It is certain, however, that in some cholera hospitals, favorably circumstanced as to size, ventilation, and space, very few of the attendants have suffered.

Of these facts we are likely to receive accurate statements, in answer to the written questions which we have submitted to the medical authorities through the government here.

Convalescence from cholera has been rapid and perfect here, as is proved by the following fact: The Minister of the Interior had given orders that all convalescents, civil as well as military, at the General Hospital, should be detained fourteen days. We inspected about two hundred of these *détenus* some days back, with Sir James Wylie, and found them in excellent health, without a single morbid sequela amongst them.

Relapses are rare in this epidemic, nor have they been often attended with fatal results: hospital servants seem to have been most liable to them. One physician had three attacks, the second severe, in which he states that he derived great benefit from the magisterium bismuthi.

In our next we shall resume the medical history of the disease, and have, &c. (Signed) WILLIAM RUSSELL, M.D.
D. BARRY, M.D.

C. C. Greville, Esq. &c.

### METEOROLOGICAL JOURNAL,

By Messrs. Harris and Co. Mathematical Instrument Makers, 50, High Holborn.

| August | Moon. | Rain gauge. | Thermom. 9 a.m. | Thermom. max. | Thermom. min. | Baro meter. 9 a.m. | Baro meter. 10 p.m. | De Luc's Hygrometer. 9 a.m. | De Luc's Hygrometer. 10p.m. | Winds. 9 a.m. | Winds. 10p.m. | Atmospheric Variation. 9 a.m. | Atmospheric Variation. 2 p.m. | Atmospheric Variation. 10 p.m. |
|---|---|---|---|---|---|---|---|---|---|---|---|---|---|---|
| 20 | | | 64 | 68 | 58 | 29.48 | 29.70 | 52 | 55 | NNW | NNE | Cloudy | Fine | Rain |
| 21 | | | 62 | 66 | 55 | .95 | 30.04 | 59 | 57 | N | N | Fine | Cloudy | Fine |
| 22 | | | 62 | 67 | 60 | 30.08 | .04 | 57 | 56 | N | NW | Cloudy | Fine | Cloud. |
| 23 | ○ | | 69 | 74 | 61 | 29.97 | 29.89 | 54 | 54 | NW | NW | Fine | — | Fine |
| 24 | | | 68 | 70 | 60 | .74 | .59 | 52 | 55 | WSW | SW | — | — | Rain |
| 25 | | | 64 | 69 | 58 | .51 | .65 | 54 | 51 | NW | W | — | — | Fine |
| 26 | | .10 | 65 | 72 | 63 | .75 | .74 | 50 | 50 | WSW | SW | — | — | — |
| 27 | | | 69 | 74 | 62 | .74 | .75 | 51 | 51 | SW | SW | — | — | Cloudy |
| 28 | | | 66 | 72 | 58 | .90 | .94 | 50 | 50 | W | WSW | — | — | Fine |
| 29 | | | 64 | 69 | 61 | 30.02 | .97 | 50 | 51 | WNW | WNW | — | — | — |
| 30 | ☾ | | 67 | 73 | 59 | 29.90 | .82 | 51 | 52 | WSW | WSW | — | — | — |
| 31 | | | 65 | 70 | 55 | .71 | .77 | 52 | 53 | W | W | — | — | — |
| *Sept.* 1 | | | 56 | 62 | 52 | .71 | .58 | 53 | 64 | NE | NE | Sleet | Rain | Rain |
| 2 | | .193 | 57 | 60 | 49 | .62 | .66 | 75 | 60 | NW | NW | Fine | Sho'ry | Fine |
| 3 | | | 56 | 62 | 50 | .70 | .66 | 52 | 59 | NNW | NW | — | Fine | — |
| 4 | | | 59 | 65 | 58 | .67 | .64 | 63 | 65 | WNW | SSW | — | Cloudy | Sho'ry |
| 5 | | | 64 | 71 | 63 | .68 | .76 | 53 | 61 | SW | WSW | Rain | Fine | Cloudy |
| 6 | ● | | 65 | 66 | 55 | .69 | .68 | 61 | 58 | WNW | SW | — | Fine | Fine |
| 7 | | | 59 | 63 | 50 | .63 | .58 | 56 | 56 | SW | S | Fine | — | Cloudy |
| 8 | | | 58 | 64 | 58 | .51 | .52 | 59 | 59 | SW | S | — | Cloudy | Rain |
| 9 | | | 61 | 65 | 56 | .52 | .50 | 57 | 57 | WSW | WNW | Cloudy | Rain | — |
| 10 | | | 60 | 63 | 52 | .63 | .73 | 60 | 60 | WNW | N | Cloudy | Fine | — |
| 11 | | | 57 | 62 | 50 | .77 | .94 | 62 | 62 | N | N | Fine | Cloudy | Fine |
| 12 | | .87 | 56 | 64 | 55 | .99 | 30.04 | 61 | 60 | N | N | Cloudy | Fine | Cloudy |
| 13 | | | 60 | 64 | 55 | 30.01 | 29.96 | 60 | 59 | NE | SE | Fine | — | — |
| 14 | ☽ | | 60 | 63 | 53 | 29.94 | .95 | 57 | 57 | SE | ENE | Cloudy | — | Fine |
| 15 | | | 57 | 61 | 56 | .97 | 30.00 | 57 | 57 | NE | NNW | Fine | — | — |
| 16 | | | 60 | 65 | 50 | 30.04 | .07 | 56 | 56 | NNE | NNE | Cloudy | — | — |
| 17 | | | 52 | 63 | 56 | .02 | 29.99 | 56 | 55 | NW | NNW | Cloudy | — | — |
| 18 | | | 61 | 65 | 54 | 29.84 | .77 | 55 | 55 | SW | SW | Fine | — | Cloudy |
| 19 | | | 63 | 66 | 56 | .73 | .62 | 58 | 59 | SSE | S | — | — | Rain |

The quantity of Rain fallen, in the month of August, was 1 inch and 82.100ths.

